# Collection of peripheral blood mononucleated cells for chronic graft-versus-host disease immunology research: safety and effectiveness of leukapheresis in 132 patients

**DOI:** 10.1186/s12967-022-03708-w

**Published:** 2022-11-08

**Authors:** Alain Mina, Lauren Curtis, Kamille West, Yu Ying Yau, Edward W. Cowen, Frances Hakim, Steven Z. Pavletic

**Affiliations:** 1 Immune Deficiency Cellular Therapy Program, Center for Cancer Research, National Cancer Institute, National Institutes of Health, 6N119, 10 Center Drive, 20892 Bethesda, MD USA; 2 Saint Agnes Cancer Institute, Baltimore, MD USA; 3 Department of Transfusion Medicine, NIH Clinical Center, National Cancer Institute, National Institutes of Health, Bethesda, MD USA; 4 Dermatology Branch, National Institute of Arthritis and Musculoskeletal and Skin Diseases (NIAMS), National Institutes of Health, Bethesda, MD USA; 5 Experimental Transplantation and Immunology Branch, National Cancer Institute, Bethesda, MD USA

**Keywords:** Apheresis, Peripheral blood mononuclear cells, Chronic graft-versus-host-disease, Transplantation

## Abstract

**Background:**

Chronic graft-versus-host disease (GVHD) is a major cause of late morbidity and non-relapse mortality in recipients of allogeneic hematopoietic cell transplantation (HCT). Its biology, however, remains poorly understood, making the studies of its biology and immunomodulatory therapies a difficult task. Such research is often hampered by lymphopenia which is common in these patients and precludes studies of critical cellular subsets across the spectrum of severity of disease. This study explores the potential of leukapheresis to safely acquire and efficiently store immune cells for immunology research in chronic GVHD.

**Methods:**

This is a cross-sectional study in which 132 consecutively accrued patients undergo optional research leukapheresis and a one-week comprehensive outpatient evaluation. Baseline clinical and laboratory data and efficiency of the procedure were reported.

**Results:**

Ninety-four of 132 patients (71%) achieved the goal collection of 2 × 10^9 PBMNCs with a mean volume processed of 4.6 L. Only mild decreases in hemoglobin, platelet, lymphocyte and monocytes were observed. All adverse events were mild (grade 1) and had resolved by the time of discharge from the apheresis unit.

**Conclusion:**

This study demonstrates feasibility, safety, and efficiency of research leukapheresis in a frail patient population. Results presented promote leukapheresis as a standard research practice option in studies of chronic GVHD in humans which may expedite advances in our understanding of this complex multisystem disease.

**Supplementary Information:**

The online version contains supplementary material available at 10.1186/s12967-022-03708-w.

## Background

Chronic GVHD is a major source of late morbidity and non-relapse mortality in patients after allogeneic hematopoietic cell transplantation for hematologic malignancies or other life threatening bone marrow diseases (allo-HCT) [1, 2]. Despite advances in transplantation practices, it continues to affect 30–50% of long-term transplant survivors [3]. The intricacies of chronic GVHD biology remain a challenge and it is not clear yet if a common pathway that would explain the entirety of its manifestations exists. Despite increasing knowledge of immunological factors that contribute to its pathophysiology [4–7], a clearer understanding of chronic GVHD will facilitate diagnosis and optimize immunomodulatory therapies and enhance clinical outcomes [5, 8, 9].

Chronic GVHD shares features with numerous autoimmune disorders, including autoantibody formation, chronic inflammatory mechanisms and systemic sclerotic manifestations, but animal models have not fully replicated human chronic GVHD and there is the prominent need for human based studies [7, 10]. Improved understanding of chronic GVHD could hinge on in depth characterization of cellular and molecular mechanisms of chronic GVHD in allo-HSCT patients. Banking of peripheral blood mononucleated cells (MNC) and sera for research has been an important step in such studies [11]. However, conventional peripheral blood draws often yield insufficient MNC for large scale immunological characterization or functional studies. Furthermore, chronic GVHD patients are frequently lymphopenic, due to immunosuppressive therapy or to the disease process itself [12, 13]. Such lymphopenia constrains studies of rare subsets of T cells or B cells, whose quantitative or functional deficits may crucially contribute to chronic GVHD [14–16]. Leukapheresis has been utilized as an alternative to blood draws, providing lymphocytes that can be either studied immediately or stored for future use in clinical therapy or research setting [17].

As part of our efforts to better understand and treat this complex disease, we explored the potential of leukapheresis to safely acquire and efficiently store immune cells for immunology research in chronic GVHD. We describe here our experience in collecting large quantities of PMNCs from 132 chronic GVHD patients at the National Institutes of Health (NIH) Clinical Center by steady-state peripheral blood leukapheresis, detailing the feasibility, clinical safety, and efficiency of this procedure in this frail patient population.

## Methods

Consecutive patients (N = 328) were enrolled between October 2004 and March 2014 on the National Cancer Institute protocol, Natural History of Chronic GVHD (NCT00092235), a cross-sectional study in which patients undergo a one-week comprehensive outpatient evaluation. Study received approval from the National Cancer Insitute institutional of review board (IRB) and patients provided written consents to undergo leukapheresis for research purposes.

Of these, 132 patients underwent optional research leukapheresis. 47 (24%) declined participation, 39 (20%) had inadequate venous access, 29 (15%) were not candidates due to age (< 18 years old), 11 (5%) had acute medical issues that precluded leukapheresis (i.e., pneumonia, positive blood cultures, orthostatic hypotension, new arrythmia) and 70 (36%) did not undergo leukapheresis because of scheduling conflicts (Table 1). An absolute lymphocyte count (ALC) cutoff of 1.0 K/µL is required at the Department of Transfusion Medicine (DTM) at the NIH to proceed with apheresis as internal data, from healthy donors, has shown low apheresis yield when ALC < 1.0 K/µL.

**Table 1:** Patient Characteristics

Characteristics	n (%) or Median
Number of eligible patients	328
Leukapheresis	132*
No Leukapheresis	196 (100)
*Lack of consent*	47 (24)
*Inadequate venous access*	39 (20)
*Pediatric (< 18)*	29 (15)
*Acute medical illness*	11 (5)
*Other (scheduling)*	70 (36)
Median Age	49.7 (range 18.2–67.7)
Gender *Male*	80 (61)
*Female*	52 (39)
Median Karnofsky performance status	80 (range 40–100)
Chronic GVHD *Severe*	82 (62)
*Moderate*	46 (35)
*Mild*	4 (3)
Mean number of organs involved	4.92
Mean lines of prior systemic treatment	3.4

*gender, performance status and chronic GVHD percentages correspond to patients who underwent leukapheresis (n = 132)

Goal yield was set at 2 × 10^9 MNC. Goal was chosen based on the expected MNC yield after a 2 L apheresis procedure from prior NIH department of transfusion medicine (DTM) experience with healthy volunteers. Unlike stem cell transplant (SCT) and Chimeric Antigen Receptor (CAR) T cell collections, a set minimum therapeutic cell target was not required.

Devices used for MNC collection were Baxter CS3000 (62 patients, 47%), Haemonetics MCS-P (18 patients, 14%) and COBE SPECTRA (52 patients, 39%). No granulocyte colony stimulating factors (G-CSF) or other stimulants were administered to the patients. Median whole blood flow rate (WBFR) was 60 ml/min (range 35–85 ml/min) and median product volume was 147 ml (range 59–450 ml) (Table 2). Median whole blood/anticoagulant (ACDA) ratio was 12:1, based on plasma requests. Common Terminology Criteria for Adverse Events (CTCAE) were graded based on review of medical records, using version 4.03 (published June 14, 2010). Leukapheresis was done on the last day of the patients’ one week visit.

**Table 2:** Patient Leukapheresis Settings

Leukapheresis data	n (%) or Median
Leukapheresis	132 (100)
Venous access *2-arm continuous flow*	108 (82)
*1-arm continuous flow*	24 (18)
Apheresis machine *Baxter CS3000*	62 (47)
*Haemonetics MC5-P*	18 (14)
*COBE Spectra*	52 (39)
Mean total run time (min)	88 (range 43–155)
Total time* (door to door, min)	155 (73–260)
Median Volume processed (L)	4.6 (0.52–5.68)
Median whole blood flow rate (mL/min)	60 (range 35–85)
Median product volume (mL)	147 (range 59–450 )

*Door to door time is defined as the duration of time from arrival to Apheresis Center to discharge

## Results

The median age of patients undergoing leukapheresis was 49.7 years (range: 18.2–67.7). The majority of patients had NIH chronic GVHD global scores that were moderate (46 patients, 35%) or severe (82 patients, 62%). Median Karnofsky performance status was 80 (range 40–100), the mean number of organs involved was 4.9 and the mean number of previous systemic treatments was 3.4 (Table 1). 51 (38%) patients had superficial/dermal sclerosis with a mean body surface area (BSA) affected of 16.8%. 52 patients (40%) had evidence of deep sclerosis with a mean BSA affected of 30.7%. 48 patients (37%) had an absolute pre-leukapheresis lymphocyte count < 1.0 K/µL. 108 (82%) patients underwent 2 arm continuous flow apheresis while 24 patients (18%) underwent one arm intermittent flow apheresis (Table 2). Nine patients (7%) underwent leukapheresis via central venous catheter while the rest via peripheral access (hand, forearm, or antecubital vein).

**Fig. 1:**
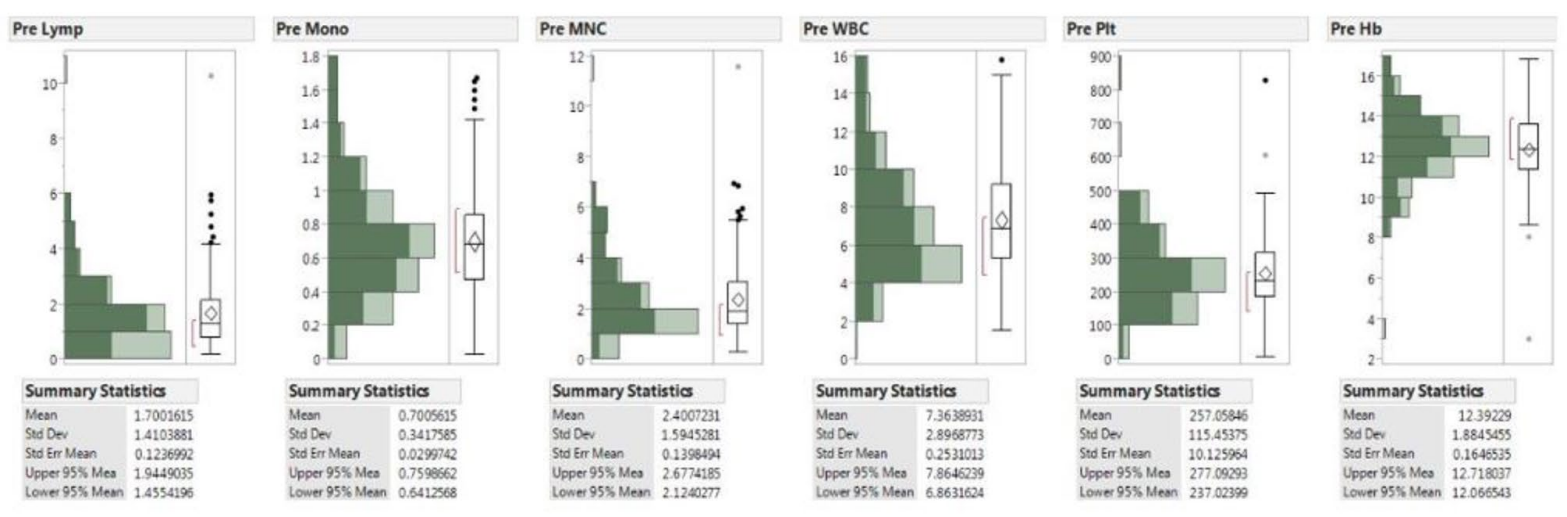
Distribution of peripheral blood cell counts in 132 chronic GVHD patients undergoing leukapheresis (dark green indicates MNC > = 2 × 10^9^).

Ninety-four of 132 patients (71%) achieved the goal collection of 2 × 10^9 PBMNCs with a mean volume processed of 4.6 L. Median total run time was 88 min. Pre-leukapheresis Hb, pre-platelet, pre-absolute lymphocytes, pre-absolute monocytes and pre-absolute MNC mean blood counts were 12.4 g/dL, 257.1 K/µL, 1.7 K/µL, 0.70 K/µL and 2.4 K/µL, respectively (Fig. 1). Collection efficiency was calculated using the following equation: CE, % = {100% x [cell content in product/((average pre and post-apheresis blood cell concentration)x(volume of blood processed))]}. Mean cell yield and efficiencies were as follows: lymphocytes: 3.7 × 10^9, 66.5%; monocytes: 1.1 × 10^9, 51.1%; granulocytes: 0.8 × 10^9, 4.5%; platelets: 1.9 × 10^11, 22.2% (Table 3). Using a previous dataset of 6578 volunteers that underwent apheresis with the same devices at the NIH, as a control cohort, efficiencies were quite comparable to those of our dataset with mean efficiencies being as follows: lymphocytes: 70.9%, monocytes: 30.6%, granulocytes: 6.1% and platelets:27.6% (See Table A in Supplemental Materials).

**Table 3:** Cell yield and collection efficiencies

	Mean cell yield	Mean efficiencies (%)
WBC		
*Lymphocyte*	3.74 × 10^9^	66.54
*Monocyte*	1.07 × 10^9^	51.12
*Granulocyte*	0.78 × 10^9^	4.54
*Platelets*	1.93 × 10 ^11^	22.24

Collection efficiency (CE, %) = {100% x [cell content in product/((average pre and post-apheresis blood cell concentration)x(volume of blood processed))]}

**Fig. 2:**
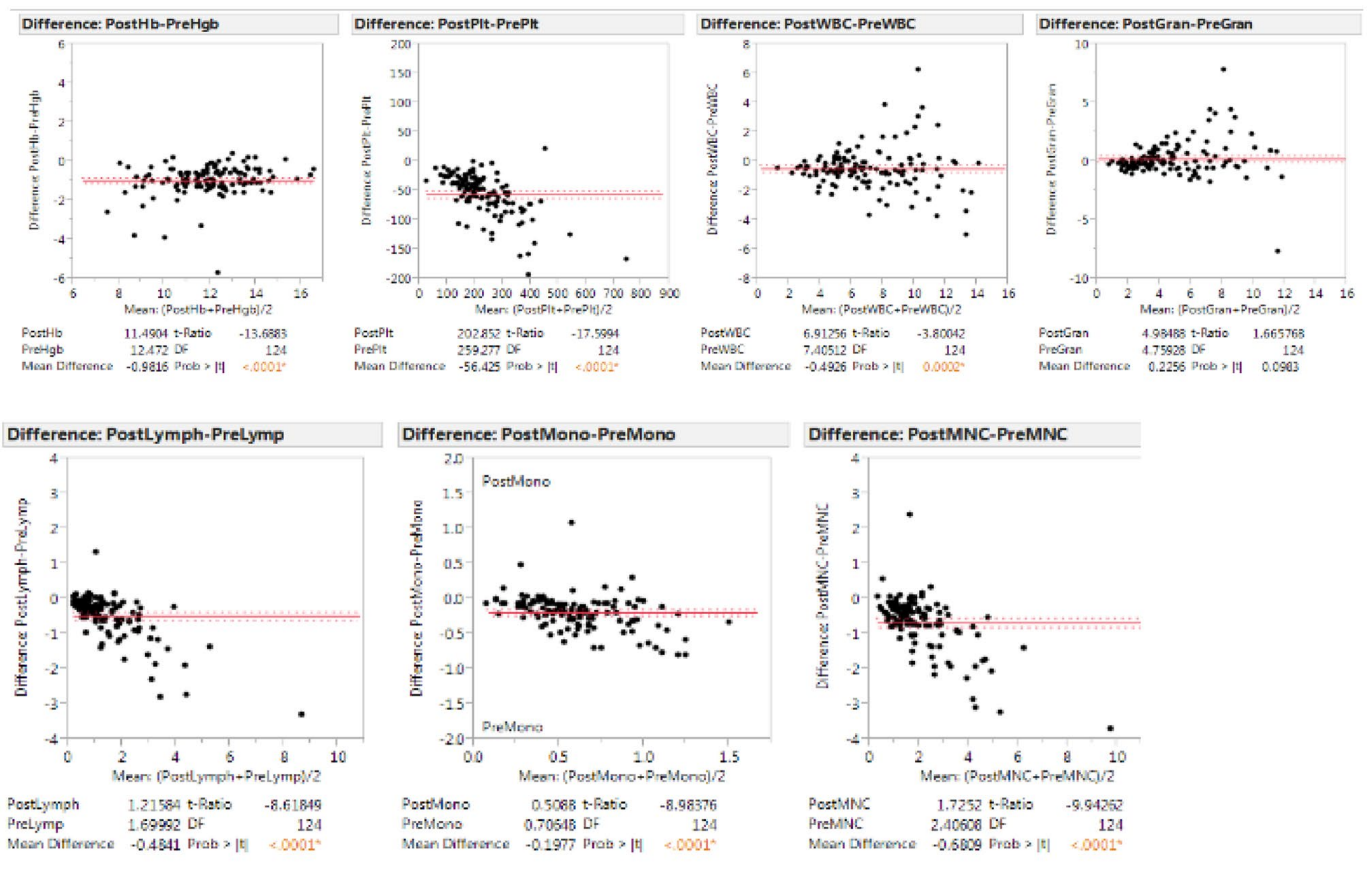
Differences in peripheral cell counts pre- and 1 h post-leukapheresis

**Fig. 3:**
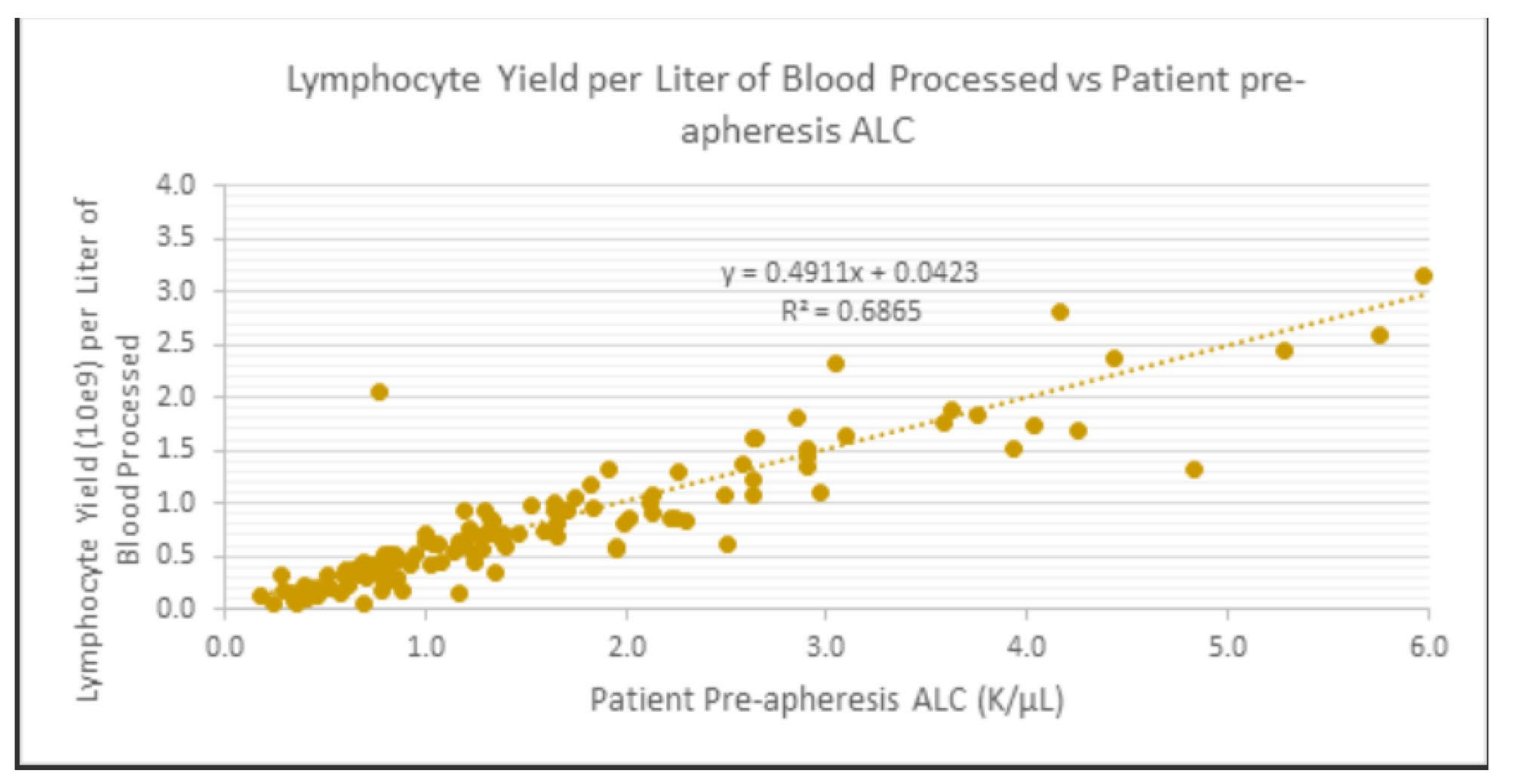
Total Lymphocyte yield per Liter of blood processed versus pre-apheresis ALC counts. Using the pre-procedure ALC (cells/µL) as the independent variables x, the collection yield (lymphocytes per Liter processed) can be estimated by the following equation: Lymph Yield/L (10^9) = 0.0423 + 0.4911*PreALC

**Fig. 4:**
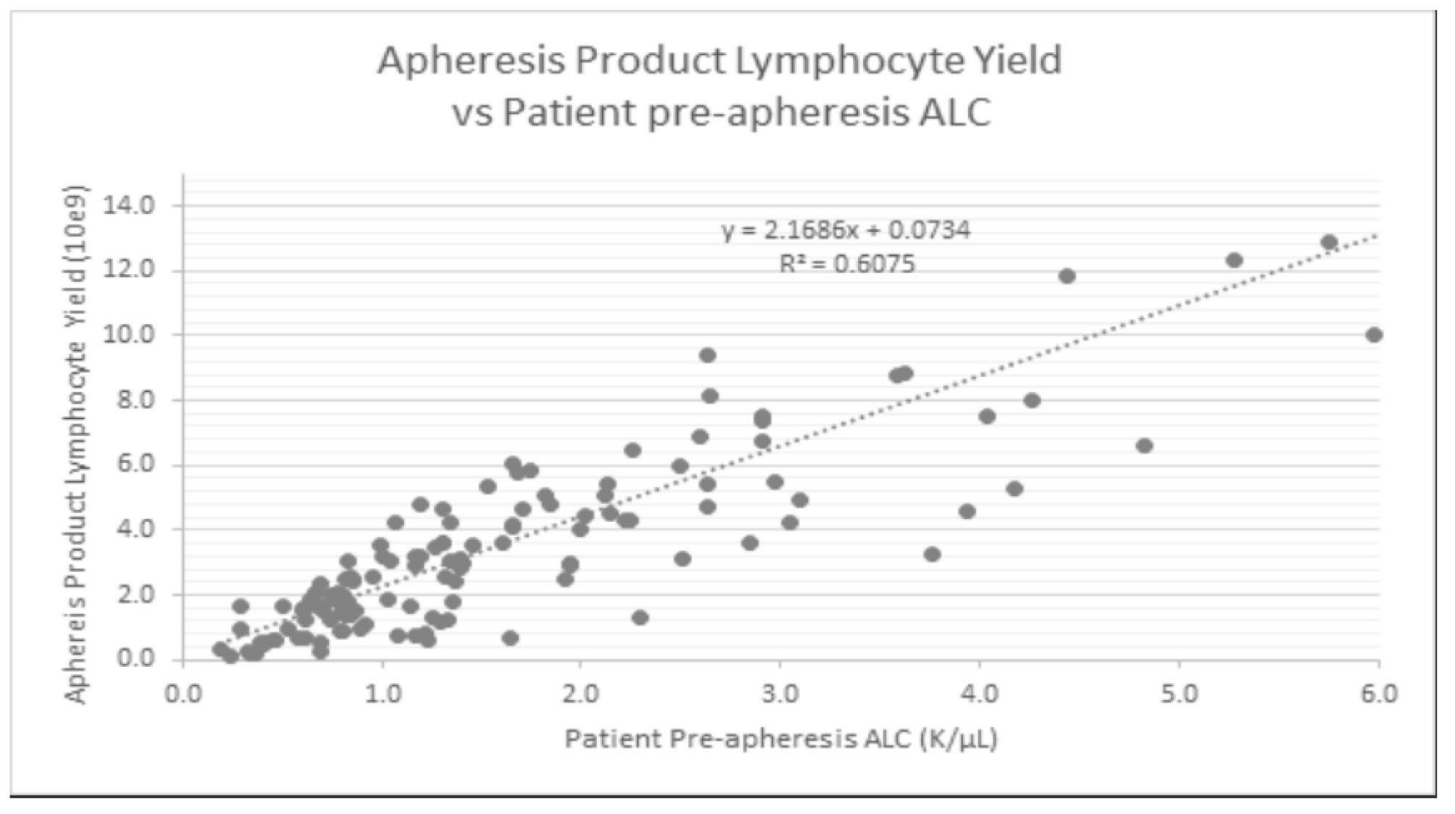
Apheresis Product Lymphocyte Yield versus Patient pre-apheresis ALC counts. Yield can be estimated by the following equation: Product Lymph Yield (10^9) = 0.0734 + 2.1686*PreALC

**Fig. 5:**
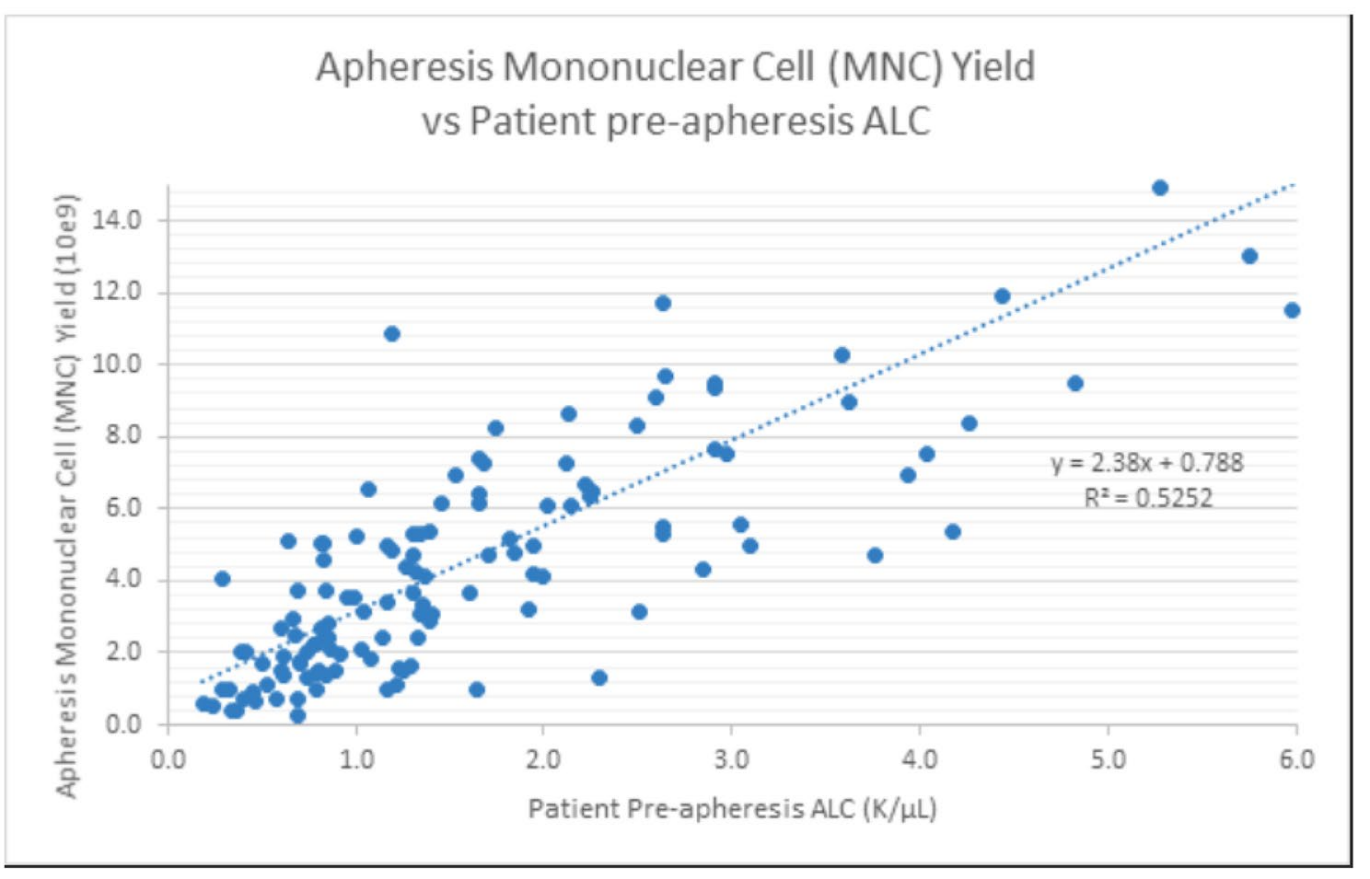
Apheresis Mononuclear Cell (MNC) Yield versus Patient pre-apheresis ALC counts. Yield can be estimated by the following equation: Product MNC Yield (10^9) = 0.0788 + 2.38*PreALC

As expected, only mild decreases in peripheral cell counts were observed (median change 1-hour post-leukapheresis): Hgb: -7.9% (p < 0.0001); platelets: -21.8% (p < 0.0001); WBC: -6.6% (p < 0.0001) absolute lymphocyte count (ALC): -28.5% (p < 0.0001); absolute monocyte count: -28.0% (p < 0.0001); MNC: -28.2% (p < 0.0001 - Fig. 2). We plotted the pre-apheresis ALC counts against the total lymphocyte yield per liter of blood processed (Fig. 3). An equation that characterizes the line that best fits the different data points can be used to prospectively guide the number of liters processed for different lymphocyte collections (Fig. 3). The pre-apheresis ALC counts could also be plotted against post-apheresis product lymphocyte yield to prospectively predict the individual efficiency of each procedure (Fig. 4). Similarly, the MNC yield could be calculated using each patient’s pre-apheresis ALC counts (Fig. 5).

All adverse events were mild (grade 1) and had resolved by the time of discharge from the apheresis unit: 1 patient had hypotension, 10 had oral dysesthesia, 6 had paresthesia, 1 had anxiety, 2 had localized bleeding and 1 had nausea (Table 4). There was no grade ≥ 2 adverse events recorded.

**Table 4:** Adverse events with leukapheresis

Adverse events (Grade 1)	n (%)
Oral dysesthesia	10 (6)
Paresthesia	6 (5)
Localized bleeding	2 (2)
Anxiety	1 (1)
Nausea	1 (1)
Hypotension	1 (1)
Total	21 (16)

## Discussion

Advances in reduced intensity conditioning regimens and in more effective antimicrobials have significantly reduced early post-transplant-related morbidity and mortality after allo-HCT. Nevertheless, late post-transplant morbidity and mortality, largely due to chronic GVHD, remains a challenge [18] The pathophysiology of chronic GVHD is complex and a better understanding of it is a must to achieving progress in its management.

Steady-state peripheral blood leukapheresis, or the collection of peripheral blood MNCs for extracorporeal photopheresis (ECP) have been used for therapeutic purposes in chronic GVHD [19–22]. Similarly, lymphapheresis is the first step of the manufacturing process of chimeric antigen receptor (CAR) T cells and has been a safe and reliable procedure for the collection of CD3 + lymphocytes from patients after allogeneic HCT [23, 24]. These procedures require a sufficient number of cells for therapeutic processing which could be a major challenge in chronic GVHD where patients are often frail or lymphopenic. Routine use of leukapheresis to acquire larger MNC numbers solely for research purposes in chronic GVHD has not been done due to concerns of logistics, feasibility and patient safety. This current study provides the evidence for the use of leukapheresis as a safe and powerful research tool for advancing knowledge about chronic GVHD and provides benchmarks for developing novel therapeutic interventions such as regulatory T cells infusion for GVHD [25] or CAR T cell therapy [26, 27].

One hundred thirty-two (40%) of the chronic GVHD patients in this study underwent research leukapheresis. Results show that independently of disease severity, extent of sclerotic disease, age or blood cell counts, steady-state PBMC leukapheresis is an overall safe, well-tolerated and effective method for large scale PBMC collection. 71% achieved the goal collection of 2 × 10^9 PBMCs, despite our GVHD population that was enriched for moderate/severe disease (97%), including many with deep sclerosis (40%). Notably, 48 patients (37%) were lymphopenic and had a pre-leukapheresis ALC < 1.0 K/µL and would be likely very difficult to study for immunological assays from conventional (20–50 ml) or large volume (250 ml) research blood draws. No patients experienced grade ≥ 2 adverse events from leukapheresis and all adverse events resolved by the time of discharge from the pheresis unit.

This report demonstrates the feasibility and safety of large scale PMNC collection and storage for research purposes and should support such practice in IRB approved chronic GVHD research protocols. As an example (see Figs. 3 and 4), if one’s research needs to use B- and/or T- lymphocytes and/or monocytes, depending on the specific project needs, it might be possible to state an arbitrary minimum lymphocyte/MNC collection target in the apheresis bag, e.g. 300 × 10e6, or 500 × 10e6, or 1.0 × 10e9 per apheresis bag. In this manner, even for the patients/procedures with the lowest pre-apheresis ALC count, a minimum of 600 × 10e6 MNCs were collected in the apheresis bag, which should be adequate for usual research assays (Fig. 2). Looking specifically at total apheresis yield and the lower range of MNC yields, 14 of 132 collections (11%) had yields of < 1.0 × 10e9 (1000 × 10e6), only 3 (2.3%) had yields of < 0.5 × 10e9 (500 × 10e6), and only 1 had yields < 0.3 × 10e9 (or 300 × 10e6), depending on what would be considered a minimum threshold for a successful or usable collection. As an example of their use in research, we and our collaborators at the NIH, were able to separate adequate numbers of FACS-selected cell populations for cell cultures and for gene expression studies relating to mechanisms of chronic GVHD [6]. Similarly, the availability of large numbers of lymphocytes using the 2 L leukapheresis permitted collaborators to analyze gene expression in cultures of sorted B cells from chronic GVHD patients; these studies identified a mechanistic link between NOTCH2 activation and robust B cell activation in chronic GVHD [28]. On the other hand, these yields might be suboptimal in the clinical setting, i.e., for therapeutic purposes. For example, CAR T cell protocols usually specify a much higher minimum number of CD3 + cells in the apheresis bag (e.g. minimum 1000 × 10e6 or 1.0 × 10e9, in order to have at least 2 aliquots of 300–500 × 10e6 CD3 + per aliquot for culture).

Patients with chronic GVHD often have decreased peripheral blood cell counts such as anemia, thrombocytopenia, neutropenia, and particularly lymphopenia, due to systemic immunosuppressive treatments or the chronic process of GVHD itself. Only mild decreases were observed in peripheral counts in this study after research leukapheresis, and no patients required blood product transfusion, colony-stimulating factors or prophylactic antibiotics. However, in person follow-up after the procedure was limited as leukapheresis was performed on the last day of their outpatient week and patients returned home soon afterwards. All patients received a follow-up phone call by a study practitioner one week after returning home, so it is possible that late onset or more durable adverse events were not adequately captured. Another limitation of our study could be the fact that our patients’ procedures were performed between 2005 and 2014 and since then, both CS300 and Spectra instruments have been retired. Newer devices such as Amicus and Optia exist. Experience with healthy volunteers in our clinic show overall lower platelet yield with Amicus and Optia. As for the healthy volunteers cohort, monocyte collection efficiency was lower using the CS-3000 than for any of the other devices (see Table A, Supplemental Materials) .

Advancing efficacy and developing personalized approaches to chronic GVHD therapy remains a major unmet need to improve long term outcomes of patients undergoing allogeneic hematopoietic cell transplantation [29]. Despite major progress in understanding the biology of chronic GVHD, further progress is needed. A better understanding of clinical – biological correlations is needed. Concerns about logistics, feasibility, and safety have been a major barrier to conducting research-directed leukapheresis to reliably obtain large numbers of PBMNCs during chronic GVHD clinical studies. Since about one third of chronic GVHD patients present with substantial lymphopenia many patients, presumably those with more severe disease [12], become excluded from immunology research studies using conventional peripheral blood draw techniques.

This study conclusively demonstrates the feasibility and safety of large scale PMNC collection and storage for research purposes and should support such practice in IRB approved chronic GVHD research protocols.

## Conclusion

In conclusion, steady-state PBMC leukapheresis is an overall safe and well-tolerated procedure in patients severely affected by chronic GVHD. Wider utilization of this approach in chronic GVHD clinical protocols should accelerate immunology research into the pathogenesis of the disease. Furthermore, this information will be useful to other research teams, grantees and funding agencies who are considering use of leukapheresis to study rare cell populations or collect PBMCs for therapeutic purposes in patients with advanced chronic GVHD. Leukapheresis should become a tool to achieve more robust research and long-awaited breakthroughs in chronic GVHD.

## Electronic supplementary material

Below is the link to the electronic supplementary material.

Supplementary Material 1

Supplementary Material 2

## Data Availability

All data generated or analyzed during this study are included in this published article.
