# Supplementary material for: Collection of peripheral blood mononucleated cells for chronic graft-versus-host disease immunology research: safety and effectiveness of leukapheresis in 132 patients
Source: J Transl Med. 2022 Nov 8;20:519. doi: 10.1186/s12967-022-03708-w (PMC9644487; doi:10.1186/s12967-022-03708-w)
Supplement: Supplementary file 1 — Supplementary Material 1 [file 12967_2022_3708_MOESM1_ESM.docx]

| **Values** | **CS3000** | **MCS-P** | **SPEC** | **Grand Total** |
| --- | --- | --- | --- | --- |
| Number of donors | 5898 | 663 | 17 | 6578 |
| Average of Lymp Eff (%) | 71.69 | 64.22 | 71.02 | 70.94 |
| Average of Mono Eff (%) | 25.78 | 72.50 | 51.44 | 30.55 |
| Average of Gran Eff (%) | 6.25 | 5.44 | 4.03 | 6.17 |
| Average of Plt Eff (%) | 26.31 | 39.28 | 23.07 | 27.61 |

**Table A: Collection efficiencies in control cohort of healthy donors by device**

| **Values** | **CS3000** | **MCS-P** | **SPEC** | **Grand Total** |
| --- | --- | --- | --- | --- |
| Number of donors | 5898 | 663 | 17 | 6578 |
| StdDev of Lymp Eff (%) | 12.99 | 11.25 | 11.37 | 13.01 |
| StdDev of Mono Eff (%) | 32.73 | 15.70 | 35.74 | 34.46 |
| StdDev of Plt Eff (%) | 10.02 | 13.08 | 5.51 | 11.07 |
| StdDev of Gran Eff (%) | 5.22 | 4.16 | 3.23 | 5.12 |

**Table B: Collection efficiencies standard deviations (StdDev) in control cohort**

**of healthy donors by device.**
