# Supplementary material for: Collection of peripheral blood mononucleated cells for chronic graft-versus-host disease immunology research: safety and effectiveness of leukapheresis in 132 patients
Source: J Transl Med. 2022 Nov 8;20:519. doi: 10.1186/s12967-022-03708-w (PMC9644487; doi:10.1186/s12967-022-03708-w)
Supplement: Supplementary file 2 — Supplementary Material 2 [file 12967_2022_3708_MOESM2_ESM.docx]

10/14/2022

Dear Editors,

We wish to submit the revised manuscript entitled “Collection of Peripheral Blood Mononucleated Cells for Chronic Graft-Versus-Host Disease Research: Safety and Effectiveness of Leukapheresis in 132 patients”. We appreciate all the excellent points that were made by the reviewers, and we have attempted to answer all their questions (see below) and make all the necessary modifications in the text.

Sincerely yours,

**Answer to reviewers’ comments:**

**Reviewer #1: Excellent study
Can the authors specify in methods if the study was reviewed by appropriate institutional review board for protection of human subjects and/if a consent process was in place for participation.**
“Study received approval from the National Cancer Institute (NCI) institutional review board (IRB) and patients provided written consents to undergo leukapheresis for research purposes.”

Text was added, tracked, in the first paragraph of the Methods’ section.

**Reviewer #2: A Mina et al. review data around non-mobilized apheresis mononuclear cell (MNC) collections from 132 patients with graft-versus-host disease to better inform the field.  The manuscript confirms the safety and feasibility/effectiveness of such collections to further study immune cells of patients with GVHD. 
I have several minor edits/comments that may improve the submission.**

**1) The calculations for efficiency are noted in Table 3.  It would be nice to see them in the text as well.**

Calculations for efficiency were added to the second paragraph of the Results section.

“…2.4 K/µL, respectively (Figure 1). Collection efficiency was calculated using the following equation: CE, % = {100% x [cell content in product/ ((average pre- and post-apheresis blood cell concentration) x (volume of blood processed))]}. Mean cell yield and efficiencies were as follows: lymphocytes: 3.7 x10^9, 66.5%; monocytes: 1.1 x10^9, 51.1%; granulocytes: 0.8 x10^9, 4.5%; platelets: 1.9 x 10^11, 22.2% (**Table 3**).”

**2) How do the efficiencies compare to any historical controls (e.g., healthy non-mob MNC donors or CART patients)?**

The following text was added to the second paragraph of the results section:

“Using a previous dataset of 6578 volunteers that underwent apheresis with the same devices at the NIH, as a control cohort, efficiencies were quite comparable to those of our dataset with mean efficiencies being as follows: lymphocytes: 70.9%, monocytes: 30.6%, granulocytes: 6.1% and platelets:27.6% (See Table A in Supplemental Materials)”.

Table A, detailing collection efficiencies in control cohort by device was added as supplemental materials.

**3) Do you note any differences among the three collection instruments?  At least two of the instruments are retired (CS300 and Spectra).  This should be noted and if you have any experience on newer instruments (e.g., Amicus, Optia), that would be helpful.**

Excellent point. The following text was added to paragraph 5 of the Discussion section.

“Another limitation of our study could be the fact that our patients’ procedures were performed between 2005 and 2014 and since then, both CS300 and Spectra instruments have been retired. Newer devices such as Amicus and Optia exist. Experience with healthy volunteers in our clinic show overall lower platelet yield with Amicus and Optia. As for the healthy volunteers cohort, monocyte collection efficiency was lower using the CS-3000 than for any of the other devices (see Table A, Supplemental Materials).”

**4) What is door to door time in Table 2?**

The following definition was added to Table 2: “Door to door time in Table 2 is defined as the duration of time from arrival to Apheresis Center to discharge.”

**5) Are you able to in general terms (i.e., without revealing prematurely your studies) describe the types of research you plan to do with the collections?  It may be interesting and add context**

Two examples of our experience with leukapheresis collection for research purposes were provided in paragraph 4 of the Discussion section as follows:

“As an example of their use in research, we and our collaborators at the NIH, were able to separate adequate numbers of FACS-selected cell populations for cell cultures and for gene expression studies relating to mechanisms of chronic GVHD[28]. Similarly, the availability of large numbers of lymphocytes using the 2L leukapheresis permitted collaborators to analyze gene expression in cultures of sorted B cells from chronic GVHD patients; these studies identified a mechanistic link between NOTCH2 activation and robust B cell activation in chronic GVHD[29].”

**6) How was the goal of 2B cells determined?  Was it the amount you wanted for specific studies?**

The following text was added to the second paragraph of the Methods’ section:

“Goal was chosen based on the expected MNC yield after a 2 liter apheresis procedure from prior NIH department of transfusion medicine (DTM) experience with healthy volunteers. Unlike stem cell transplant (SCT) and Chimeric Antigen Receptor (CAR) T cell collections, a set minimum therapeutic cell target was not required.”

**7) In Table 4 it would be nice to have some reference to patients that had more than 1 AE.  So, a way to see what percent of patients had an AE?  Were AEs more likely in the severe groups versus moderate and mild?**

Thank you for this suggestion. We checked and only a few patients had more than one adverse event therefore a too small number to make any kind of association with severity of chronic GVHD or any other variable.

**8) In Methods, end of 1st paragraph, is the 1.0K/uL cut=off for healthy donors only?  Or was it determined using healthy donors?  Clarifying this further would be helpful, as you did collect patients below that cut-off.**

Excellent point. A cutoff point of 1.0K/uL is used in our healthy donors’ protocols. No cutoffs were used for our patients. The following text was added to the second paragraph of the Methods’ section

“An absolute lymphocyte count (ALC) cutoff of 1.0 K/µL is required at the Department of Transfusion Medicine (DTM) at the NIH to proceed with apheresis as internal data, from healthy donors, has shown low apheresis yield when ALC < 1.0K/µL.”

**9) In Results, 2nd paragraph, you may have flipped the pre-abs lymph and pre-abs mono counts based on the "respectively".**

Thank you. Error is now rectified in text.

**10) Brief mention of the efficiency calculation may be best in the 2nd paragraph of Results where you mention mean cell yield and efficiencies.**

Efficiency calculation is now added to 2^nd^ paragraph of Results section.

**11) In Table 1, the '100' after 132, should be 40% if you want to include percentage (132/328).  In the same table you could delete the '%' after 80 and 52 since you indicate above that percents will be in parentheses.  And, finally, adding range for bottom two numbers would be more complete.**

The number “328” refers to the total number of eligible patients that were enrolled on the National Cancer Institute protocol, Natural History of Chronic GVHD (NCT00092235). “132” is the total number of patients, out of the “328” that underwent the procedure of leukapheresis. Gender, performance status and chronic GVHD percentages correspond to patients who underwent leukapheresis (n=132). Explanation is now added under Table 1 and percentages were modified in text.

**12) In Table 2, you can delete 'mL' at the bottom after 147 since it is included in the first column.**

Comment is now addressed in the text.

**13) In Table 3, the superscripts are low.**

Comment is now addressed in the text.

**14) In Figure 4, in the title, should '/L' be deleted since this is absolute count and not per Liter?  Same for Figure 5?**

Comments are now addressed in the text.

**15) Page 5, line 58: It states that "14 of 143" collections, but previous section says the total is 132. Please review this.**

Thank you again. Comment is now addressed in the text.
